# Three-dimensional laparoscopic Roux-en-Y gastric bypass with totally hand-sewn anastomoses for morbid obesity. A single center experience^1^

**DOI:** 10.1590/s0102-865020200080000006

**Published:** 2020-09-07

**Authors:** Francesco Mongelli, Davide La Regina, Fabio Garofalo, Alberto Vannelli, Matteo Di Giuseppe, Maurice FitzGerald, Michele Marengo

**Affiliations:** I MD, Department of Surgery, Ospedale Regionale di Lugano, Lugano, Switzerland. Scientific, intellectual, conception and design of the study; analysis and interpretation of data; statistics analysis; manuscript writing; final approval.; II MD, Department of Surgery, Ospedale Regionale di Bellinzona e Valli, Bellinzona, Switzerland. Analysis and interpretation of data, manuscript preparation, critical revision, final approval.; III MD, Department of Surgery, Ospedale Regionale di Lugano, Lugano, Switzerland. Critical revision, final approval.; IV MD, Department of Surgery, Ospedale Valduce, Como, Italy. Analysis and interpretation of data, critical revision, final approval.; V MD, Department of Surgery, Ospedale Regionale di Bellinzona e Valli, Bellinzona, Switzerland. Acquisition of data, manuscript preparation, critical revision, final approval.; VI MD, Department of Surgery, Ospedale Regionale di Bellinzona e Valli, Bellinzona, Switzerland. Acquisition of data, statistics analysis, manuscript writing, final approval.; VII MD, Department of Surgery, Ospedale Regionale di Bellinzona e Valli, Bellinzona, Switzerland. Scientific, intellectual, conception and design of the study, technical procedures, manuscript writing, final approval.

**Keywords:** Gastric Bypass, Laparoscopy, Bariatric Surgery, Imaging, Three-Dimensional, Operative Time

## Abstract

**Purpose:**

To assess the impact of three-dimensional (3D) vision use on operative time (OT) in laparoscopic Roux-en-Y gastric bypass (LRYGB) with hand-sewn anastomoses.

**Methods:**

We analyzed a prospectively collected database of patients who underwent LRYGB. We included all patients operated on with either 2D or 3D vision. Demographics and clinical characteristics, operative time, hospital stay and 30-day postoperative complications were collected for all patients and analyzed.

**Results:**

During the study time, out of 143 patients who underwent LRYGB for morbid obesity, 111 were considered eligible. Seventy-eight patients were operated with 2D vision and 33 patients with 3D vision. Demographics and clinical characteristics were not different among groups. Mean OT was 203±51 and 167±32 minutes in the 2D and 3D groups respectively (p<0.001). Multivariate analyses showed that increasing age and BMI were independently related to prolonged OT, while 3D vision (OR 6.675, 95% CI 2.380-24.752, p<0.001) was strongly associated with shorter OT.

**Conclusions:**

The use of 3D vision in LRYGB significantly reduced the OT, though intra- and postoperative complication rates and the length of hospital stay were not affected. Despite its limitations, our study supports the value of 3D vision laparoscopy in bariatric surgery.

## Introduction

Laparoscopic Roux-en-Y gastric bypass (LRYGB) for morbid obesity is one of the most frequently performed bariatric interventions worldwide^1^. This procedure requires surgical prowess and adequate experience^2^ in order to contain operative time (OT) and avoid complications^3,4^. According to current evidence, a higher BMI leads to a longer operative time. In addition, the latter seems to have an impact on postoperative complications^5,6^.

Today, most surgeons still use two-dimensional (2D) laparoscopy, which technology provides a flat-field image, unfavorable in terms of depth perception and hand-eye coordination^7^. The development of surgically applied three-dimensional (3D) stereoscopic vision provides a better depth perception and improves hand-eye coordination. Such advantages are particularly relevant when performing complex laparoscopic tasks such as tissue manipulation and dissection, suturing and knot tying^7^. On the other hand, differences are insignificant while performing basic laparoscopic skills^7,8^.

The aim of our study was to compare the OT in LRYGBs performed with either 2D or 3D vision by experienced bariatric surgeons.

## Methods

### *Study design*

We retrospectively analyzed a prospectively collected database on patients who underwent a LRYGB for morbid obesity from January 2014 to April 2018. Written informed consent was obtained before the collection of data and the local ethical committee approved the study.

Patients undergoing revision bariatric interventions or concomitant procedures such as cholecystectomy and bowel adhesiolysis, were excluded. Demographic data were collected and recorded in a database: age, sex, co-morbidities (obstruction sleep apnea syndrome, arterial hypertension, type 2 diabetes mellitus), body mass index (BMI), length of hospital stay, overall OT, intraoperative complications and 30-day complication rate (Clavien-Dindo classification)^9^.

All operations were performed with either 2D (Richard Wolf Treier, Full HDTV 3CCD, LMD-2450MD) or 3D vision (Braun Aesculap EinsteinVision 2.0, 3D Full HD, Monitor 32”). The chronological intraoperative use of 2D vs. 3D was mixed, according to the availability of the surgical instruments and never related to the surgeons’ choice. The primary end-point was the overall OT, secondary end-points were hospital stay and intra- and postoperative complication rates.

### *Surgical technique*

With either 2D or 3D vision, the lesser omentum was initially divided and an estimated 20 ml gastric pouch was created with a 30 mm and 45 mm linear stapler (Endo GIA™, Medtronic). The alimentary limb was measured to a length of 100-150 cm according to the BMI and the bowel divided with a 45 mm linear stapler. The jejunojejunal side-to-side anastomosis was created with a running 2-0 Vicryl suture and reinforced with 2-0 polyester (Ethibond) suture. Subsequently, hand-sewn ante-colic gastro-jejunostomy was performed with 2 layers of 2-0 polyglactin (Vicryl) suture. The hydropneumatic (leak-test) test was performed to assess anastomotic permeability. Mesenteric defects and Petersen’s space were closed with 2-0 polyester (Ethibond) suture.

### *Statistical analysis*

Based on previous studies^8,10^, the estimated sample size with 80% power and 0.05 significance was 48 patients (24 per group). Dichotomous variables were expressed as absolute frequencies and the Chi-squared test was used for the group analysis. Continuous variables were expressed as mean with standard deviation (SD) and compared with the Student t-test. Univariate and multivariate analyses with odds ratio (OR) and 95% confidence interval (CI) were performed to test association between the above reported variable of interest. The used statistical software was MedCalc Statistical Software version 19.1.5 (MedCalc Software bvba, Ostend, Belgium; http://www.medcalc.org; 2017).

## Results

During the study time, out of 143 patients who underwent LRYGB for morbid obesity, 111 were considered eligible for the study. Seventy-eight patients were operated with 2D vision and 33 patients with 3D vision. Thirty-five (32%) patients were male, mean age was 44.6 ± 11.3 years and mean BMI was 43.8 ± 6.1 kg/m^2^. Demographics and clinical characteristics were not different among groups (Table 1).

**Table 1:** Patients’ demographics and clinical characteristics.

	2D vision n=78	3D vision n=33	p
Male, n (%)	25 (32)	10 (30)	0.896
Age, years (SD)	44.7 (11.3)	44.3 (11.6)	0.859
BMI, kg/m^2^ (SD)	44.1 (6.1)	43.2 (6.2)	0.461
OSAS, n (%)	29 (37)	9 (27)	0.476
Arterial hypertension, n (%)	22 (28)	7 (21)	0.554
Diabetes mellitus, n (%)	10 (13)	5 (15)	0.776

Continuous variables are expressed as mean with standard deviation (SD) in parentheses. Categorical variables are reported

as absolute number with frequencies in parentheses. BMI = Body mass index, OSAS = Obstruction sleep apnea syndrome.

The mean OT was 203 ± 51 and 167 ± 32 minutes in the 2D and 3D groups respectively (p<0.001). During the surgical intervention four complications occurred: in the 2D group, a case of inadequate dimension of the gastric pouch required a resizing, a case of tension on the gastro-jejunal anastomosis required a conversion to open surgery and a case of liver injury was resolved with hemostatics. In the 3D group only a case of ileal resection due to iatrogenic injury near to the proximal anastomosis was recorded (Table 2). Defining prolonged OT as longer the mean value, univariate and multivariate analyses showed that increasing age (OR 1.055, 95% CI 1.011-1.101, p=0.015) and BMI (OR 1.146, 95% CI 1.057-1.243, p=0.001) were independently related to prolonged OT, while 3D vision (OR 6.675, 95% CI 2.380-24.752, p<0.001) was strongly associated with shorter OT (Table 3).

**Table 2:** Primary and secondary outcomes.

	2D vision n=78	3D vision n=33	p
Operative time, min (SD)	203 (51)	167 (32)	<0.001
Intraoperative complications, n (%)	3 (4)	1 (3)	0.839
Hospital stay, days (SD)	7.1 (1.1)	6.3 (0.7)	<0.001
Postoperative complications, n (%)	4 (5)	1 (3)	0.642

Continuous variables are expressed as mean with standard deviation (SD) in parentheses. Categorical variables are reported

as absolute number with frequencies in parentheses.

**Table 3:** Uni and multivariate analyses of factors associated with prolonged operative time.

	Short operative time (n=65)	Prolonged operative time (n=46)	Univariate analysis (OR and 95% CI)	Multivariate analysis (OR and 95% CI, Forward, α=0.1)	p
Age (years)			**1.032 (0.997-1.069)**	**1.055 (1.011-1.101)**	**0.015**
Gender (n)					
Female	51	25	1		
Male	14	21	**3.060 (1.336-7.007)**	2.579 (0.967-6.879)	0.058
BMI (kg/m^2^)			**1.112 (1.037-1.192)**	**1.146 (1.057-1.243)**	**0.001**
Surgical intervention					
Laparoscopy					
2D vision	37	41	1		
3D vision	28	5	**0.161 (0.056-0.461)**	**0.130 (0.040-0.420)**	**<0.001**
Intraoperative complications					
No	65	42	1		
Yes	1	3	4.465 (0.449-44.357)		0.201
Postoperative course					
Complications Clavien-Dindo ≥ 3					
No	55	49	1		
Yes	1	4	6.095 (0.658-56.438)		0.111

Values are presented as absolute numbers. The variables included in the multivariate analysis are marked in bold. OR = odds ratio, CI = confidence interval.

Postoperatively, five (4.5%) complications Clavien-Dindo ≥ 3 were recorded: in the 2D group the patients who needed the conversion to open surgery required a re-laparotomy to exclude bowel ischemia, an anastomotic leakage of the jejuno-jejunal anastomosis and two cases of postoperative bleeding required a re-laparoscopy. In the 3D group a patient developed a gastrointestinal bleeding due to an anastomotic ulcer on the 8^th^ postoperative day and was treated endoscopically. The mean length of hospital stay was 7.1 ± 1.1 and 6.3 ± 0.7 days in the 2D and 3D groups respectively (p<0.001). Defining prolonged length of hospital stay as values ≥ 7.0 days, uni- and multivariate analyses showed that OT (OR 1.021, 95% CI 1.007-1.035, p=0.004) was the only factor independently associated to a prolonged hospital stay (Table 4).

**Table 4:** Uni and multivariate analyses of factors associated with prolonged hospital stay.

	Short hospital stay (n=88)	Prolonged hospital stay (n=23)	Univariate analysis (OR and 95% CI)	Multivariate analysis (OR and 95% CI, Forward, α=0.1)	p
Age (years)			0.996 (0.957-1.038)		0.867
Gender (n)					
Female	63	13	1		
Male	25	10	1.938 (0.753-4.991)		0.156
BMI (kg/m^2^)			1.015 (0.943-1.093)		0.685
Surgical intervention					
Laparoscopy					
2D vision	56	22	1		0.064
3D vision	32	1	**0.079 (0.010-0.618)**	0.135 (0.016-1.122)	
Intraoperative complications					
No	84	21			
Yes	2	2	4.095 (0.545-30.786)		0.171
Operative time			**1.022 (1.009-1.034)**	**1.021 (1.007-1.035)**	**0.004**
Postoperative course					
Complications Clavien-Dindo ≥ 3					
No	84	20			
Yes	2	3	**6.450 (1.009-41.194)**	3.606 (0.344-37.775)	0.284

Values are presented as absolute numbers. The variables included in the multivariate analysis are marked in bold. OR = odds ratio, CI = confidence interval.

## Discussion

In our study, the use of 3D vision in LRYGB for morbid obesity was strongly associated to shorter OTs in the multivariate analysis, without any effect on intra- and postoperative complication rates and length of hospital stay.

LRYGB requires advanced laparoscopic skills and can be technically demanding^2^, especially in patients with very high BMI, in which case both operative time and complication rate are reported to be higher^3-5^. Standardize operative techniques and modern devices have been developed to contain OT. Among others, mechanical stapler anastomoses and simplified interventions such as one-anastomosis gastric bypass are to be mentioned^11,12^; nonetheless, LRYGB remains the standard of treatment^13^. According to surgeon and center experience, different anastomosis techniques are used in LRYGB (linear or circular staplers, hand-sewn technique)^14^. Despite being mechanical anastomoses technically easier and operatively faster, recent studies have described a lower rate of wound infection and postoperative bleeding in case of hand-sewn anastomoses, with a leak rate comparable to the mechanical ones^2,14,15^. In our series, the overall complications rate was 4.5%, close to that reported in the literature, without difference between 2D and 3D groups^2,16^.

In 3D laparoscopy, depth perception and hand-eye coordination are improved, resulting in remarkable advantages while carrying out determined tasks such as tissue manipulation and dissection, suturing and knot tying^8,17-20^. On the contrary, no clear advantages in using 3D laparoscopy have been reported while performing simpler surgical tasks, such as trocar insertion, intraperitoneal exploration and identification of common structures^18^. Surgeons with early experience in minimally invasive techniques do not compensate for the lack of field depth yet and benefit from 3D laparoscopy^21^. For experienced surgeons, no reduction in OT has been described in easy or common surgical operations (i.e. laparoscopic cholecystectomies), in contrast to their junior colleagues that do not^20,22^. Conversely, in challenging laparoscopic operations such as gastrectomy, lymphadenectomies and colon resections the 3D vision has shown advantages for both experienced and novice surgeons^23^. The above-mentioned rationale is expected to help in reducing OT, which is independent risk factor for adverse outcomes in a wide range of surgical procedures, including laparoscopic Roux-en-Y gastric bypass^24-26^. In addition, in the presence of increasing financial pressure, surgeons are today expected to contain their OT while ensuring a low complication rate^27^. In our study cohort, 2D and 3D groups resulted to be similar in terms of demographics, clinical characteristics and complications. For what concerns the OT, a positive effect was noted after introducing the 3D vision. Moreover, totally hand-sewn LRYGB was subjectively performed with notable advantages in the execution of complex surgical tasks (i.e. manipulation and dissection of tissues, suture of mesenteric defects and bowel anastomoses). In bariatric surgery, Currò *et al*.^8^ and Rojano-Rodríguez *et al*.^28^ described similar impressions.

We measured a 203 ± 51 minutes long OT for 2D LRYGB with hand-sewn anastomoses, similar to the one reported by Acín-Gándara *et al*.^2^, but longer to the ones described by Finks *et al*.^29^ and Awad *et al*.^16^ (127.1 ± 50.4 and 127 ± 30 minutes respectively). To test the reliability of our findings and to eliminate confounders, multivariate analyses were carried out. Factors associated with a prolonged operative time were age, BMI and 2D vision. Male gender was also associated to a prolonged OT, although it did not reach the statistical significance. Age, BMI and gender are expected to influence OTs as they are related to the adipose tissue excess and its distribution within the body. The longer OT in our series may be explained by performing both anastomoses (gastro-jejunal and jejuno-jejunal) in hand-sewn technique, while generally the jejuno-jejunal is performed with staplers. Regarding the hospital stay, even if a significant difference was noted between groups with the Student t-test, the multivariate analysis did not demonstrate any correlation with the 3D vision.

The use of 3D vision for prolonged operations has been criticized due to the onset of several side-effects: headache, eye strain, disorientation, dizziness and discomfort^22^, which often precluded its use in long operations. However, the rapid improvements of the 3D equipment have shown an equally rapid decrease in the number and the severity of subjective side-effects^30^, a finding confirmed by our surgeons.

This study has some limitations. First, the lack of randomization, even if the retrospective analysis of a prospectively collected dataset should minimize the experimenter-expectancy effect^31^. Exact records of the intra-operative time dedicated to perform each hand-sewn anastomosis using 2D or 3D vision would have been of interest. On the contrary, we recorded the overall OTs, which may vary from patient to patient depending on a range of factors such as anatomy, BMI, intraoperative complications and concomitant procedures. However, we excluded patients that underwent other planned procedures to analyze comparable OTs. Particularly interesting would be a cost-effectiveness analysis as the actual time saved during the surgical intervention should reduce costs as well. Finally, the number of patients was limited and larger studies are required to better define advantages of 3D vision in LRYGB.

## Conclusions

The use of 3D vision in LRYGB with totally hand-sewn anastomoses significantly reduced the OT, though intra- and postoperative complication rates and the length of hospital stay were not affected. Despite its limitations, our study supports the value of 3D vision laparoscopy in bariatric surgery.
